# Chitin-Derived Nitrogen-Doped Carbon Nanopaper with Subwavelength Nanoporous Structures for Solar Thermal Heating

**DOI:** 10.3390/nano13091480

**Published:** 2023-04-26

**Authors:** Thanakorn Yeamsuksawat, Luting Zhu, Takaaki Kasuga, Masaya Nogi, Hirotaka Koga

**Affiliations:** SANKEN (The Institute of Scientific and Industrial Research), Osaka University, 8-1 Mihogaoka, Ibaraki 567-0047, Osaka, Japan; y.thanakorn@eco.sanken.osaka-u.ac.jp (T.Y.); sharollzhu@eco.sanken.osaka-u.ac.jp (L.Z.); tkasuga@eco.sanken.osaka-u.ac.jp (T.K.); nogi@eco.sanken.osaka-u.ac.jp (M.N.)

**Keywords:** chitin nanofiber, biomass-derived carbon, subwavelength nanoporous structures, nitrogen-doped carbon, solar thermal heating

## Abstract

Sustainable biomass-derived carbons have attracted research interest because of their ability to effectively absorb and convert solar light to thermal energy, a phenomenon known as solar thermal heating. Although their carbon-based molecular and nanoporous structures should be customized to achieve enhanced solar thermal heating performance, such customization has insufficiently progressed. In this study, we transformed a chitin nanofiber/water dispersion into paper, referred to as chitin nanopaper, with subwavelength nanoporous structures by spatially controlled drying, followed by temperature-controlled carbonization without any pretreatment to customize the carbon-based molecular structures. The optimal carbonization temperature for enhancing the solar absorption and solar thermal heating performance of the chitin nanopaper was determined to be 400 °C. Furthermore, we observed that the nitrogen component, which afforded nitrogen-doped carbon structures, and the high morphological stability of chitin nanofibers against carbonization, which maintained subwavelength nanoporous structures even after carbonization, contributed to the improved solar absorption of the carbonized chitin nanopaper. The carbonized chitin nanopaper exhibited a higher solar thermal heating performance than the carbonized cellulose nanopaper and commercial nanocarbon materials, thus demonstrating significant potential as an excellent solar thermal material.

## 1. Introduction

Solar thermal heating has been receiving increasing attention from researchers as a promising process for using solar light as thermal energy. Solar thermal heating requires photothermal materials, which absorb light and convert it into heat [1,2]. Examples of photothermal materials include plasmonic metal nanoparticles, metal oxide semiconductors, carbon materials [2], and plasmonic metamaterials [3,4]. Among these, carbon materials exhibit a broad light absorption range [5], which covers the wavelength range of solar light (300–2500 nm, ASTM G173-03, Air Mass 1.5 Global spectrum (AM1.5G) [6]). Biomass-derived carbons have been used as sustainable photothermal materials for a variety of solar thermal heating applications, such as solar steam generation [7], desalination, wastewater purification [8,9], and photothermal catalysis [10].

The rational structural design of biomass-derived carbons is desirable for further enhancing their solar thermal heating performance [2]. For instance, carbon-based molecular structures, including sp^2^-hybridized carbon and heteroatom-doped carbon structures with a customized distribution of the highest occupied molecular orbital and lowest unoccupied molecular orbital, should be designed. The design of such carbon-based molecular structures can influence not only light absorption but also the conversion of the absorbed light to heat by vibration relaxation [11,12]. Furthermore, subwavelength nanoporous structures should be designed to suppress light reflection and facilitate light absorption via light confinement, as reported for plasmonic metal nanoparticles and metal oxide semiconductors [13].

We previously reported that the carbon material derived from wood cellulose nanofiber paper, referred to as cellulose nanopaper, whose molecular and subwavelength nanoporous structures can be customized, acts as a photothermal material for solar thermal heating [14]. The carbon-based molecular structures of cellulose nanopaper were customized by controlling the carbonization temperatures; the sp^2^-hybridized carbon structures formed by semicarbonization at 500 °C afforded high solar light absorption by adequately balancing solar light absorption and reflection. Subwavelength nanopore structures have also been constructed within cellulose nanopaper-derived carbon by expanding the pore spaces between cellulose nanofibers via treatment with low-surface-tension *tert*-butyl alcohol (*t*-BuOH) [15], thereby suppressing solar light reflection. The resulting cellulose nanopaper-derived carbon with customized molecular and subwavelength nanoporous structures exhibited effective solar thermal heating performance, which was higher than those of previously reported biomass-derived carbons and conventional nanocarbon materials, including graphene and carbon nanotube films [14]. However, cellulose nanopaper requires iodine gas pretreatment (100 °C) for a long duration (24 h) to retain its customized subwavelength nanoporous structures after carbonization because the morphology of cellulose nanofibers collapses after high-temperature treatment [15]. Hence, discovering alternative biomass nanofibers that can retain their original morphology without pretreatment, even after carbonization, with adequate carbon-based molecular structures for excellent solar thermal heating is desirable.

Chitin (β-(1→4)-linked *N*-acetyl anhydroglucosamine) is among the most abundant biomass materials on earth. Its molecular structure is similar to that of cellulose, except for the presence of an acetyl-amino group instead of a hydroxyl group on C-2 in cellulose. Chitin can be extracted as nanofibers from the exoskeletons of crustacean wastes, such as crabs, squid pens, and prawns [16,17,18,19]. Chitin nanofibers intrinsically contain nitrogen derived from their acetyl-amino group, providing an opportunity to prepare N-doped carbon structures by carbonization [20,21]. N-doped carbon structures have demonstrated enhanced functionality in various applications such as energy storage [20,22,23], adsorption and catalysis [21], photosensing [23], and microwave absorption [24]. Moreover, chitin nanofibers exhibit higher thermal stability than cellulose nanofibers against the collapse of their morphology during carbonization [25]. Thus, carbonized chitin nanofibers could be a superior alternative to carbonized cellulose nanofibers as high-performance photothermal materials. However, to the best of our knowledge, the photothermal heating properties of carbonized chitin nanofiber materials have not yet been explored.

In this study, chitin-nanofiber-derived N-doped carbon was fabricated and evaluated as a photothermal material for solar thermal heating. Chitin nanofibers were transformed into a nanopaper with subwavelength nanoporous structures by expanding the pore spaces between the nanofibers via *t*-BuOH treatment. Subsequently, carbonization was performed without pretreatment at controlled temperatures to customize the carbon-based molecular structures. Moreover, to elucidate the significance of the carbonized chitin nanopaper, its solar thermal heating performance was compared with that of a carbonized cellulose nanopaper.

## 2. Materials and Methods

### 2.1. Materials

Aqueous dispersions of chitin nanofibers (2 wt%, SFo-20002) and cellulose nanofibers (2 wt%, WFo-10002) were obtained from Sugino Machine Ltd., Namerikawa, Japan. *t*-BuOH (>99% purity) was supplied by Nacalai Tesque Inc., Kyoto, Japan.

### 2.2. Preparation and Carbonization of Cellulose and Chitin Nanopapers

A water dispersion of chitin or cellulose nanofibers (0.2 wt%, 200 mL) was vacuum filtered on a hydrophilic polytetrafluorethylene membrane (pore diameter: 0.2 μm, H020A090C, Advantec Toyo Kaisha, Ltd., Tokyo, Japan). The nanofibers on the membrane were then treated by gently pouring *t*-BuOH onto it while vacuum filtering. The resulting wet nanopaper was peeled from the membrane, stored in a refrigerator (SJ-23T-S, Sharp, Corp., Osaka, Japan) at −18 °C for 0.5 h, and then freeze-dried overnight (FDU-2200, Tokyo Rikakikai Co., Ltd., Tokyo, Japan). Subsequently, the as-prepared nanopaper with a thickness of ~300 μm was cut into a square sheet with an area of 1.5 × 1.5 cm^2^. A molybdenum block (area: 1.5 × 1.5 cm^2^, thickness: ~1.0 cm, weight: ~20 g, MO-293771, The Nilaco Corp., Tokyo, Japan) was placed on it. Then, carbonization was performed in a furnace (KDF-75, DENKEN-HIGHDENTAL Co., Ltd., Kyoto, Japan) under a N_2_ gas flow at a flow rate of ~500 mL min^−1^ in three stages [14]: (1) the temperature was increased from room temperature to 240 °C at 2 °C min^−1^ and maintained for 17 h; (2) the temperature was increased from 240 °C to the target temperature (300–1100 °C) at 2 °C min^−1^ and maintained for 1 h; and (3) the temperature was decreased to room temperature at 2 °C min^−1^.

### 2.3. Solar Thermal Heating Performances

Following our previous study [14], we evaluated the solar thermal heating performances of the original and carbonized nanopapers by measuring the changes in their surface temperatures during solar light irradiation. Prior to the evaluation, the emissivity of each nanopaper was evaluated using a commercial black tape (emissivity: 0.95, HB-250, OPTEX Co., Ltd., Otsu, Japan) as a reference. Briefly, black tape and a carbonized nanopaper were heated to 75 °C on a thermo-controller (SBX-303, Sakaguchi E.H VOC Corp., Tokyo, Japan). The emissivity of each nanopaper was estimated using a thermal imaging camera (FLIR ETS320, FLIR Systems. Inc., Wilsonville, OR, USA) by adjusting the temperature according to the reference temperature of black tape. Subsequently, surface temperature measurements were performed using a solar simulator (HAL-320W, Asahi Spectra Co., Ltd., Tokyo, Japan). The nanopaper with an area of less than 1.5 × 1.5 cm^2^ was placed on an acrylic plate (3 × 3 cm^2^) with a rectangular hole (0.7 × 0.7 cm^2^). Thereafter, it was irradiated by simulated solar light (AM1.5G, light intensity: 1.0 kW m^−2^ (1 sun)) such that the area of light illumination was larger than that of the nanopaper. The surface temperature of the nanopaper was recorded using a thermal imaging camera, and its equilibrium surface temperature was evaluated from the average temperature during a solar illumination time of 500–600 s (~850 plots). More than five samples were prepared and evaluated under each condition. The surface temperature measurements were conducted at 25 °C and 65% relative humidity.

### 2.4. Optical Properties

The light absorption, transmittance, and reflection of the carbonized chitin and cellulose nanopapers were evaluated using an ultraviolet−visible−near-infrared (UV–vis–NIR) spectrometer (UV-3600i Plus, Shimadzu Corp., Kyoto, Japan) equipped with an ISR-603 integrating sphere (Shimadzu Corp., Kyoto, Japan). More than five samples were prepared and evaluated under each condition. Light absorption was calculated from the total light transmittance and reflection spectra. Solar absorption was calculated using Equation (1) [26] as follows:

(1) $$\bar{\alpha }\;\left(\%\right)=\frac{\int_{\lambda_{min}}^{\lambda_{max}}I_{solar}\left(\lambda \right)\cdot \alpha_{solar}\left(\lambda \right)d\lambda }{\int_{\lambda_{min}}^{\lambda_{max}}I_{solar}\left(\lambda \right)d\lambda }\times 100,$$

where $\bar{\alpha }$ is the solar absorption (%); *λ* is the wavelength (nm)*; λ_min_* and *λ_max_* are 300 and 2500 nm, respectively; *I_solar_*(*λ*) is the solar spectral irradiance (AM1.5G) at *λ*; and *α_solar_*(*λ*) is the light absorption (%) at *λ*. The optical bandgap values were also calculated from the UV–vis–NIR absorption spectra according to a previously reported method [15] and Tauc’s equation [27] (Equation (2)):

(*αhν*)^1/*n*^ = *A*(*hν* −*E*_g_), (2)

where α, *hν*, *A*, and *E*_g_ are the absorbance, photon energy, constant, and optical band gap, respectively. The optical bandgap was estimated by plotting (*αhν*)^1/*n*^ vs. photon energy (*hν*) and extrapolating the linear region of the curve to the *X*-axis (Figure S1). The parameter *n* was set to 2 for the indirect transition of the carbonized nanopapers because of their amorphous carbon structures.

### 2.5. Molecular Structures

Laser Raman spectroscopic analyses were performed using a RAMAN-touch VISNIR-OUN spectrometer (Nanophoton Corp., Osaka, Japan) with an incident laser wavelength of 532 nm. Elemental analyses were performed using a 2400II instrument (PerkinElmer Japan Co., Ltd., Kanagawa, Japan). X-ray photoelectron spectroscopy (XPS) profiles were recorded using a JPS-9010 photoelectron spectrometer with a monochromatic Al Kα X-ray source (1486.6 eV) (JEOL, Ltd., Tokyo, Japan) at 15 kV voltage and 20 mA current.

### 2.6. Nanoporous Structures

Surface structures of the original and carbonized chitin and cellulose nanopapers were observed using field-emission scanning electron microscopy (FE-SEM) (SU-8020, Hitachi High-Tech Science Corp., Tokyo, Japan) at an accelerating voltage of 2 kV. Prior to FE-SEM, platinum sputtering of the samples was conducted using an E-1045 Ion Sputter (Hitachi High-Tech Science Corp., Tokyo, Japan) at a current of 20 mA for 10 s. Pore size distribution curves were obtained using nitrogen adsorption analysis at −196 °C based on the Brunauer−Emmett−Teller and density functional theory models (NOVA 4200e, Quantachrome Instruments, Kanagawa, Japan).

## 3. Results and Discussion

**Figure 1:**
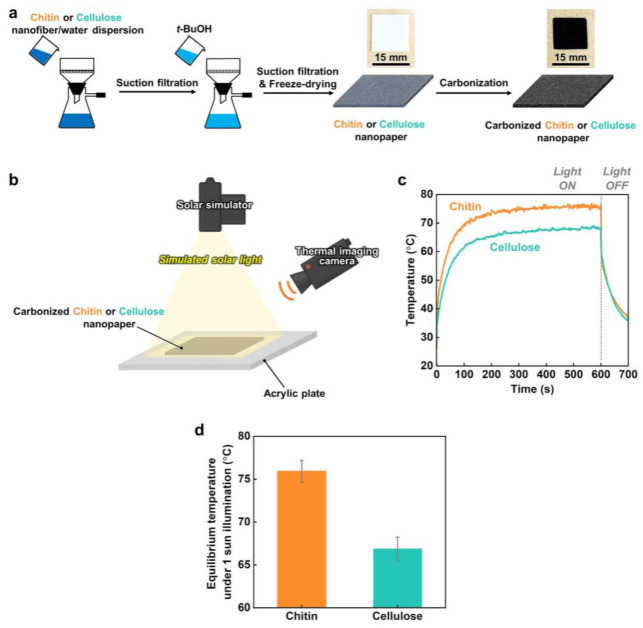
Preparation and solar thermal heating performance of carbonized chitin and cellulose nanopapers. (**a**) Schematic of the preparation of the original and carbonized chitin or cellulose nanopaper and optical images of the original and carbonized chitin nanopaper; (**b**) schematic of the experimental setup for the measurement of the surface temperature during simulated solar light illumination; (**c**) surface temperature evolution and (**d**) equilibrium surface temperature of carbonized chitin and cellulose nanopapers under 1-sun illumination. Carbonization temperature: 400 °C.

The fabrication of the carbonized chitin nanopaper is schematically illustrated in Figure 1a. A water dispersion of crab-shell-derived chitin nanofibers (0.2 wt%, 200 mL) was suction filtered. Then, it was treated by gently pouring *t*-BuOH (200 mL) onto the resulting wet sheet and vacuum filtered. Next, it was freeze-dried overnight and carbonized at 400 °C under a N_2_ atmosphere without any pretreatment. The color of the chitin nanopaper changed from white to black after carbonization. Similarly, a carbonized cellulose nanopaper was fabricated using a water dispersion of wood-derived cellulose nanofibers (0.2 wt%, 200 mL). Although the chitin and cellulose nanopapers became somewhat brittle after carbonization, they were freestanding and allowed easy handling for characterization and evaluation.

The solar thermal heating properties of the carbonized chitin and cellulose nanopapers were evaluated and compared. As shown in Figure 1b, the change in the surface temperature of the carbonized chitin or cellulose nanopaper under simulated solar illumination (AM1.5G, light intensity: 1 sun) was monitored using a thermal imaging camera. The surface temperatures of the carbonized chitin and cellulose nanopapers rapidly increase upon 1-sun illumination and are saturated within 600 s (Figure 1c). The equilibrium surface temperatures of the original chitin and cellulose nanopapers (before carbonization) are 37.7 ± 0.40 and 37.1 ± 1.40 °C, respectively, while those of the carbonized chitin and cellulose nanopapers are 75.9 ± 1.27 and 66.9 ± 1.40 °C, respectively (Figure 1d), suggesting that the carbonized chitin nanopaper exhibits a higher solar thermal heating performance than the carbonized cellulose nanopaper.

**Figure 2:**
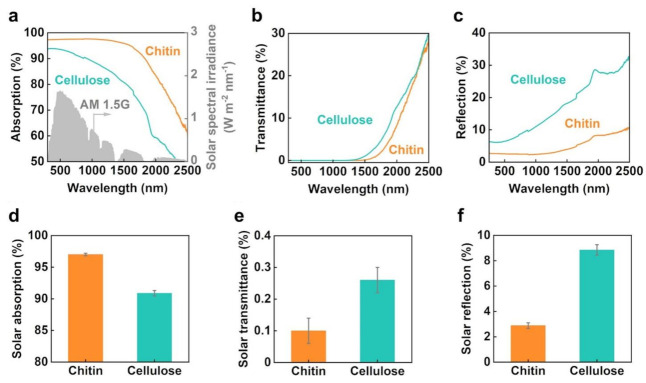
Light absorption properties of the carbonized chitin and cellulose nanopapers. (**a**) AM1.5G solar spectral irradiance and UV−vis−NIR absorption, (**b**) transmittance, and (**c**) reflection spectra; solar light (**d**) absorption, (**e**) transmittance, and (**f**) reflection. Carbonization temperature: 400 °C.

Solar thermal heating by photothermal materials depends on their ability to absorb solar light and convert it into heat [1,2]. Therefore, to observe the difference in the solar thermal heating properties of the carbonized chitin and cellulose nanopapers, their light absorption properties were compared (Figure 2). As shown in Figure 2a, the carbonized chitin nanopaper exhibits a higher light absorption than the carbonized cellulose nanopaper in the wavelength range of solar light (AM1.5G, 300–2500 nm) [6]. The higher light absorption of the carbonized chitin nanopaper is attributed to (1) the light transmission (transmittance: ~0%) wavelength being extended to a longer wavelength region (Figure 2b) and (2) light reflection being suppressed in the entire solar wavelength region (300−2500 nm) (Figure 2c). Furthermore, for a clearer comparison of the carbonized chitin and cellulose nanopapers, their solar absorptions, transmittances, and reflections were calculated from UV−vis−NIR absorption, transmittance, and reflection spectra, respectively, and the AM1.5G solar spectral irradiance, according to a previously reported method [26]. As shown in Figure 2d–f, the carbonized chitin nanopaper provides a higher solar absorption (97.0% ± 0.19%) than the carbonized cellulose nanopaper (90.9% ± 0.43%) owing to the slight suppression of solar transmittance and large suppression of reflection. Thus, the carbonized chitin nanopaper exhibits a higher solar thermal heating performance than the carbonized cellulose nanopaper owing to higher solar absorption.

**Figure 3:**
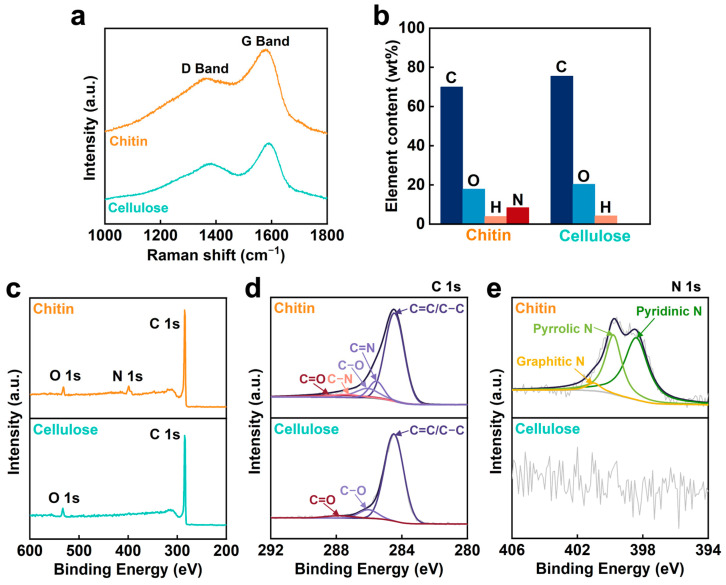
Molecular structures of the carbonized chitin and cellulose nanopapers. (**a**) Raman spectra, (**b**) element contents, (**c**) wide XPS profiles, (**d**) C 1s XPS, and (**e**) N 1s XPS. Carbonization temperature: 400 °C.

The UV–vis–NIR absorption and transmission spectra of the carbonized chitin nanopaper exhibit light absorption over longer wavelength regions compared to those of the carbonized cellulose nanopaper (Figure 2a,b). This characteristic of the carbonized chitin nanopaper is beneficial for the suppression of light transmission and reflection in the longer wavelength region. We attributed this phenomenon to the lower optical bandgap of the carbonized chitin nanopaper (0.74 eV) compared with that of the carbonized cellulose nanopaper (1.01 eV) (see Supplementary Information, Figure S1). A lower bandgap can facilitate light absorption at lower energies (longer wavelengths). To confirm that the carbonized chitin nanopaper exhibited a lower optical bandgap, its molecular structures were analyzed and compared with those of the carbonized cellulose nanopaper (Figure 3). The original chitin and cellulose nanopapers before carbonization have the wide σ−σ* bandgap due to their sp^3^-hybridized carbon structures, resulting in low light absorption (i.e., high light transmission and reflection) (Figure S2). The Raman spectra of the carbonized chitin and cellulose nanopapers display G and D bands (Figure 3a), which are associated with graphitic sp^2^-hybridized carbon domains and defective carbon structures [28], respectively, indicating that both chitin and cellulose nanopapers formed graphitic and defective carbon structures after carbonization at 400 °C. Owing to the graphitic carbon structures (i.e., π-orbital), the carbonized chitin and cellulose nanopapers had the π−π* bandgap, which lies within the σ−σ* bandgap. Hence, the carbonized nanopapers could promote light absorption as compared with the original nanopapers, while suppressing light transmission and reflection (Figure 2a–c and Figure S2). Elemental analyses show that the carbonized chitin nanopaper contains C (69.9 wt%), O (17.8 wt%), H (3.90 wt%), and N (8.40 wt%) and that the carbonized cellulose nanopaper contains C (75.5 wt%), O (20.3 wt%), and H (4.20 wt%) (Figure 3b). The presence of N in the carbonized chitin nanopaper was verified by XPS (Figure 3c). The C 1s spectrum of the carbonized chitin nanopaper can be deconvoluted into five peaks: C–C or C=C (284.6 eV), C=N (285.8 eV), C–O (286.1 eV), C–N (287.4 eV), and C=O (287.8 eV) [29,30], whereas that of the carbonized cellulose nanopaper shows three peaks: C–C or C=C, C–O, and C=O (Figure 3d). The N 1s spectrum of the carbonized chitin nanopaper suggests the formation of pyridinic N (398.4 eV), pyrrolic N (399.9 eV), and graphitic N (401.0 eV) [31] (Figure 3e). These results indicate that the carbonized cellulose nanopaper possessed O-doped defective carbon structures, whereas the carbonized chitin nanopaper possessed N- and O-doped defective carbon structures. The carbonized cellulose nanopaper had graphitic sp^2^-hybridized carbon domains (*π*-orbital) and defective regions such as O-containing functional groups (*n*-orbital), in which the *n* energy level lies within the *π*–π* energy gaps and reduces the optical bandgap [32]. The carbonized chitin nanopaper had additional N-containing functional groups (*n*-orbital) in the defective regions, which could further reduce the optical bandgap. Thus, the N- and O-doped defective carbon structures of the carbonized chitin nanopaper result in a decreased optical bandgap, facilitating light absorption at lower energies (longer wavelengths) and consequently enhancing its solar absorption performance.

**Figure 4:**
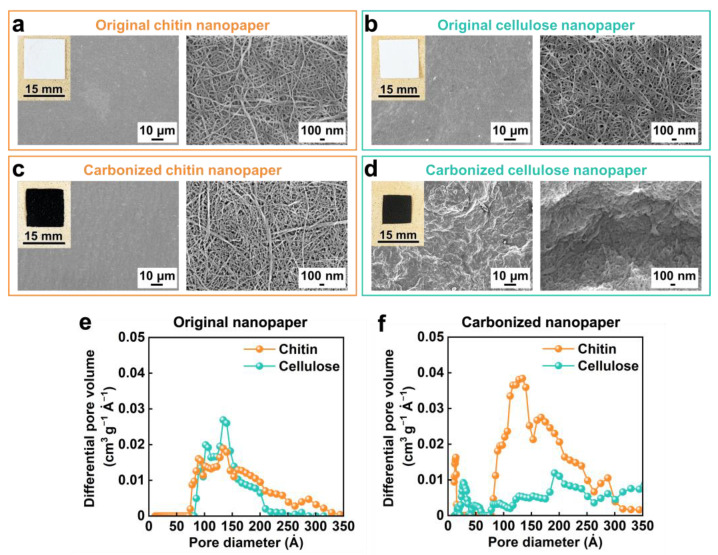
Morphologies of the carbonized chitin and cellulose nanopapers. Optical and field-emission scanning electron microscopy images of the original (**a**) chitin and (**b**) cellulose nanopapers and the carbonized (**c**) chitin and (**d**) cellulose nanopapers; pore size distribution curves of the (**e**) original and (**f**) carbonized chitin and cellulose nanopapers. Carbonization temperature: 400 °C.

Unlike the carbonized cellulose nanopaper, the carbonized chitin nanopaper suppresses light reflection over the entire solar wavelength region (Figure 2c), demonstrating excellent solar absorption. To determine the reason for this suppressed light reflection, the morphologies of the carbonized chitin and cellulose nanopapers were analyzed and compared (Figure 4). The original chitin and cellulose nanopapers prepared in this study constitute subwavelength nanoporous structures that can suppress light reflection via the light confinement effect [13]. The subwavelength nanoporous structures of both nanopapers have similar pore size distributions (Figure 4a,b,e). However, the cellulose nanopaper shrinks considerably after carbonization, closing its nanopores and forming microscale wrinkles on its surface (Figure 4d). The resulting dense and microscale structures cause light reflection [14]. By contrast, the shrinkage in chitin nanopaper after carbonization is lower than that in the cellulose nanopaper, which helps maintain the nanoporous structure and prevents the formation of microscale wrinkles in chitin nanopaper (Figure 4c,f). The morphological stability of the chitin nanopaper against carbonization could be attributed to the acetyl-amino group of chitin [33]. Hence, the carbonized chitin nanopaper demonstrated the light confinement effect owing to its subwavelength nanoporous structures, which suppress light reflection, thereby facilitating solar absorption.

**Figure 5:**
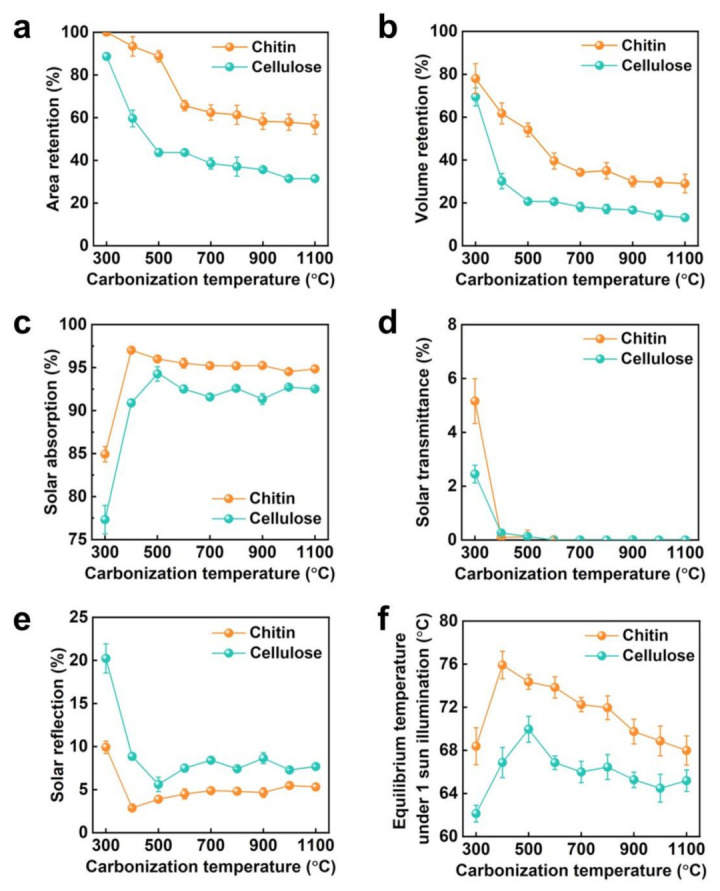
Morphology retention, solar absorption properties, and solar thermal heating performances of the chitin and cellulose nanopapers carbonized at different temperatures. (**a**) Area and (**b**) volume retention; solar (**c**) absorption, (**d**) transmittance, and (**e**) reflection; and (**f**) equilibrium surface temperature under 1-sun illumination. Carbonization temperature: 300−1100 °C.

Finally, the morphologies and solar thermal heating properties of the carbonized chitin and cellulose nanopapers were compared at different carbonization temperatures. At the carbonization temperatures of 300–1100 °C, the chitin nanopaper retains more area and volume than the cellulose nanopaper (Figure 5a,b), thus retaining the subwavelength nanoporous structures even at 1100 °C (Figure S3). Furthermore, the carbonized chitin nanopaper suppresses solar reflection and demonstrates higher solar absorption than does the carbonized cellulose nanopaper at all carbonization temperatures (300−1100 °C) (Figure 5c–e). Although the thicknesses of the carbonized chitin and cellulose nanopapers were gradually decreased with increasing carbonization temperatures (Figure S4), the carbonized nanopapers exhibited very low solar transmittance, regardless of their carbonization temperatures (Figure 5d). These results suggested that the thickness of the carbonized nanopapers investigated in this study is not a dominant factor for their solar absorption properties. Notably, the chitin nanopaper carbonized at 400 °C exhibits the highest solar absorption and the highest equilibrium surface temperature under 1-sun illumination (Figure 5c,f). The solar absorption and surface temperature under 1-sun illumination of the carbonized chitin nanopaper gradually decrease with increasing carbonization temperature above 400 °C. The lower solar absorption at higher carbonization temperatures (Figure 5c) is attributed to higher light reflection (Figure 5e); the light reflection increases with increasing carbonization temperatures, possibly due to the gradual growth of graphitic sp^2^-hybridized carbon domains by removing N and O [23,24]. The increased light reflection could be derived from a graphitic carbon domain-induced metallic luster, as reported for carbonized cellulose nanopaper [14], graphite films [34], and graphene papers [35]. The gradual formation of microscale wrinkles on the surfaces of the carbonized chitin nanopaper with increasing carbonization temperatures (Supplementary Information, Figure S3a) could also increase its light reflection. The lower surface temperature under 1-sun illumination at higher carbonization temperatures could be attributed to the increased through-plane thermal conductivity resulting from the growth of the graphitic sp^2^-hybridized carbon domain [14] in addition to the decreased solar absorption. The optimal carbonization temperature for solar absorption and surface temperature under 1-sun illumination are slightly different for the chitin (400 °C) and cellulose nanopapers (500 °C) (Figure 5c,f), which could be ascribed to the balance of their carbon-based molecular structures and morphologies. Moreover, the chitin nanopaper exhibits the best solar thermal heating performance at a lower carbonization temperature of 400 °C than that of the cellulose nanopaper (500 °C). Furthermore, the carbonized chitin nanopaper exhibits a higher solar thermal heating performance than the carbonized cellulose nanopaper regardless of the carbonization temperature. The solar thermal heating performance (surface temperature under 1-sun illumination: 75.9 ± 1.27 °C) of the chitin nanopaper carbonized at 400 °C is superior to those of commercial nanocarbon materials, such as carbon nanotube black body (55.0 °C), graphite sheet (64.5 °C), graphene paper (65.2 °C), and graphene oxide film (69.4 °C) [14].

## 4. Conclusions

In this study, chitin and cellulose nanopapers were prepared and carbonized. The carbonized chitin nanopaper demonstrated excellent solar thermal heating properties owing to its N-doped carbon structures and subwavelength nanoporous structures, which facilitated effective solar thermal heating. The optimal carbonization temperature for the chitin nanopaper was 400 °C, which balanced its carbon-based molecular structure and nano/microscale morphology. The solar thermal heating performance of the optimized carbonized chitin nanopaper (solar absorption: 97.0 ± 0.19%, surface temperature under 1 sun illumination: 75.9 ± 1.27 °C) was higher than that of the carbonized cellulose nanopaper (solar absorption: 94.3 ± 0.85%, surface temperature under 1-sun illumination: 69.9 ± 1.20 °C). This was attributed to the lower optical bandgap in the carbonized chitin nanopaper derived from its N-doped carbon structures and higher morphological stability against carbonization that maintained its subwavelength nanoporous structures without any pretreatment. Thus, carbonized chitin nanopaper is expected as a promising photothermal material towards the effective use of solar energy. Further design of nanoporous structures within carbonized chitin nanopaper can enhance its solar thermal heating performance. Moreover, the strategies presented herein can promote the functionalization of carbonized bionanofiber materials with heteroatom-doped and nanostructured carbons for various applications.

## Data Availability

Data presented in this study are available in this article.

## Supplementary Materials

The following supporting information can be downloaded at: https://www.mdpi.com/article/10.3390/nano13091480/s1, Figure S1: Tauc plots and estimated optical bandgap values of chitin and cellulose nanopapers carbonized at 400 °C; Figure S2: Light absorption properties of the original chitin and cellulose nanopapers before carbonization; Figure S3: Morphologies of chitin and cellulose nanopapers carbonized at 500 and 1100 °C; Figure S4: Thickness of the chitin and cellulose nanopapers carbonized at different temperatures.
