# Supplementary material for: Chitin-Derived Nitrogen-Doped Carbon Nanopaper with Subwavelength Nanoporous Structures for Solar Thermal Heating
Source: Nanomaterials (Basel). 2023 Apr 26;13(9):1480. doi: 10.3390/nano13091480 (PMC10179754; doi:10.3390/nano13091480)
Supplement: Supplementary file 1 [file nanomaterials-13-01480-s001.zip › nanomaterials-2328692-supplementary.pdf]

nanomaterials

Supplementary Materials

Chitin-Derived Nitrogen-Doped Carbon Nanopaper with Subwavelength Nanoporous Structures for Solar Thermal Heating

Thanakorn Yeamsuksawat, Luting Zhu, Takaaki Kasuga, Masaya Nogi and Hirotaka Koga *

SANKEN (The Institute of Scientific and Industrial Research), Osaka University, 8-1 Mihogaoka, Ibaraki, Osaka 567-0047, Japan; y.thanakorn@eco.sanken.osaka-u.ac.jp (T.Y.); sharollzhu@eco.sanken.osaka-u.ac.jp (L.Z.); tkasuga@eco.sanken.osaka-u.ac.jp (T.K.); nogi@eco.sanken.osaka-u.ac.jp (M.N.)
* Correspondence: hkoga@eco.sanken.osaka-u.ac.jp; Tel.: +81-6-6879-8442; Fax: +81-6-6879-8444

Supporting Figures

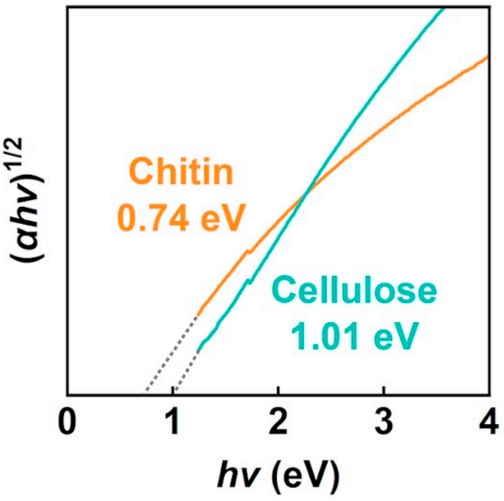

Figure S1. Tauc plots and estimated optical bandgap values of the carbonized chitin and cellulose nanopapers. Carbonization temperature: 400 °C.

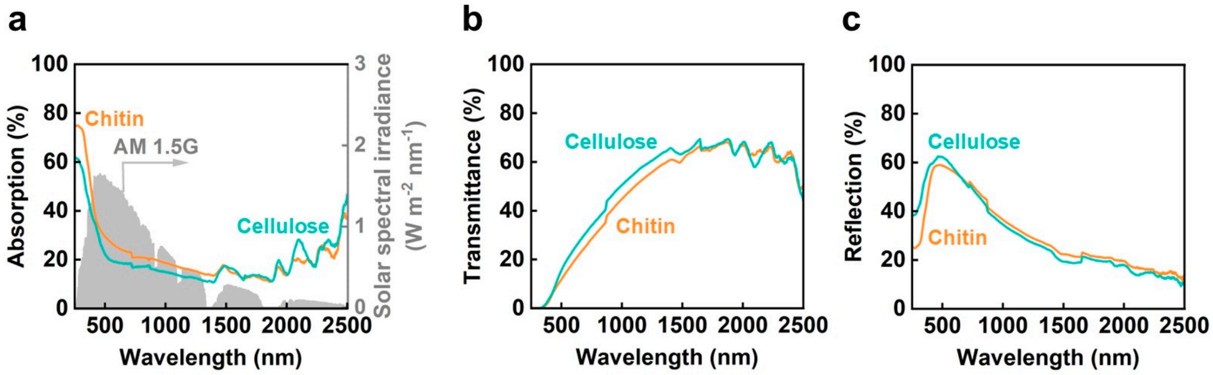

Figure S2. Light absorption properties of the original chitin and cellulose nanopapers before carbonization. (**a**) Solar spectral irradiance (AM1.5G) and UV−vis−NIR absorption, (**b**) transmittance, and (**c**) reflection spectra.

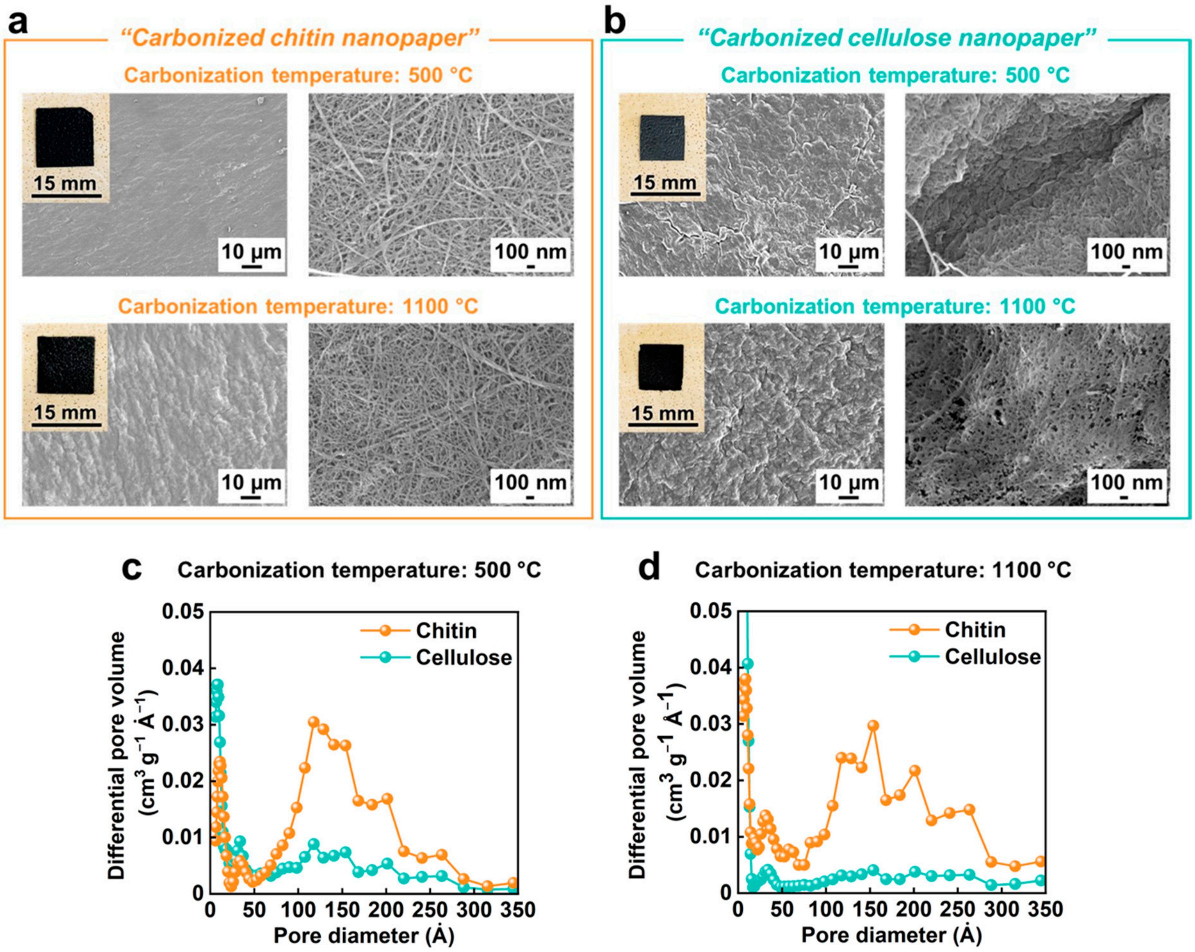

Figure S3. Morphologies of the carbonized chitin and cellulose nanopapers. Optical and field-emission scanning electron microscopy images of the carbonized (**a**) chitin and (**b**) cellulose nanopapers, and pore size distribution curves of the chitin and cellulose nanopapers carbonized at (**c**) 500 and (**d**) 1100 °C. Carbonization temperature: 500 or 1100 °C.

The carbonized chitin nanopaper could retain the subwavelength nanoporous structures even at 1100 °C, while microscale wrinkles were gradually formed on its surfaces with increasing carbonization temperatures.

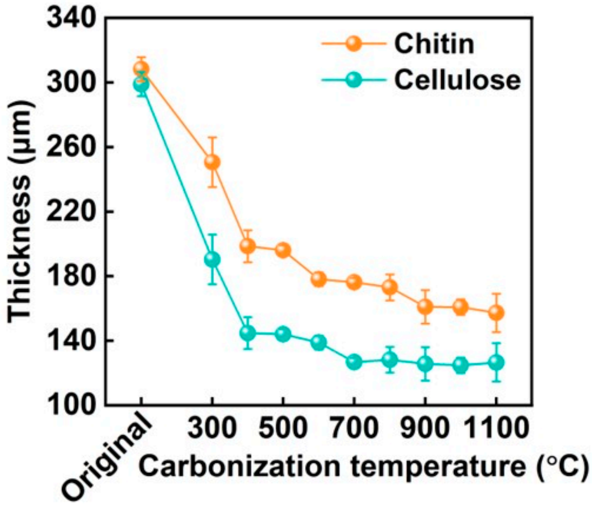

Figure S4. Thickness of the chitin and cellulose nanopapers carbonized at different temperatures.
